# Angptl4 is upregulated under inflammatory conditions in the bone marrow of mice, expands myeloid progenitors, and accelerates reconstitution of platelets after myelosuppressive therapy

**DOI:** 10.1186/s13045-015-0152-2

**Published:** 2015-06-09

**Authors:** Anne Schumacher, Bernd Denecke, Till Braunschweig, Jasmin Stahlschmidt, Susanne Ziegler, Lars-Ove Brandenburg, Matthias B. Stope, Antons Martincuks, Michael Vogt, Dieter Görtz, Annalisa Camporeale, Valeria Poli, Gerhard Müller-Newen, Tim H. Brümmendorf, Patrick Ziegler

**Affiliations:** Department of Oncology, Hematology and Stem Cell Transplantation, University Hospital Aachen, RWTH Aachen University, Pauwelsstrasse 30, 52074 Aachen, Germany; Interdisciplinary Center for Clinical Research IZKF Aachen, RWTH Aachen University Hospital, Aachen, Germany; Institute of Pathology, University Hospital Aachen, RWTH Aachen University, Pauwelsstrasse 30, 52074 Aachen, Germany; Department of Anatomy and Cell Biology, RWTH Aachen University, Wendlingweg 2, 52074 Aachen, Germany; Department of Urology, University Medicine Greifswald, Greifswald, Germany; Department of Biochemistry and Molecular Biology, University Hospital Aachen, RWTH Aachen University, Pauwelsstrasse 30, 52074 Aachen, Germany; Institute for Laboratory Animal Science, University Hospital, Pauwelsstrasse 30, 52074 Aachen, Germany; Department of Molecular Biotechnology and Health Sciences, Molecular Biotechnology Center, University of Turin, 10126 Turin, Italy; Institute for Occupational and Social Medicine, Aachen University, Aachen, Germany

**Keywords:** Inflammatory conditions, Angptl4, Platelet reconstitution, Myelosuppressive therapy, STAT3

## Abstract

**Background:**

Upon inflammation, myeloid cell generation in the bone marrow (BM) is broadly enhanced by the action of induced cytokines which are produced locally and at multiple sites throughout the body.

**Methods:**

Using microarray studies, we found that Angptl4 is upregulated in the BM during systemic inflammation.

**Results:**

Recombinant murine Angptl4 (rmAngptl4) stimulated the proliferation of myeloid colony-forming units (CFUs) in vitro. Upon repeated in vivo injections, rmAngptl4 increased BM progenitor cell frequency and this was paralleled by a relative increase in phenotypically defined granulocyte-macrophage progenitors (GMPs). Furthermore, in vivo treatment with rmAngptl4 resulted in elevated platelet counts in steady-state mice while allowing a significant acceleration of reconstitution of platelets after myelosuppressive therapy. The administration of rmAngptl4 increased the number of CD61^+^CD41^low^-expressing megakaryocytes (MK) in the BM of steady-state and in the spleen of transplanted mice. Furthermore, rmAngptl4 improved the in vitro differentiation of immature MKs from hematopoietic stem and progenitor cells. Mechanistically, using a signal transducer and activator of transcription 3 (STAT3) reporter knockin model, we show that rmAngptl4 induces de novo STAT3 expression in immature MK which could be important for the effective expansion of MKs after myelosuppressive therapy.

**Conclusion:**

Whereas the definitive role of Angptl4 in mediating the effects of lipopolysaccharide (LPS) on the BM has to be demonstrated by further studies involving multiple cytokine knockouts, our data suggest that Angptl4 plays a critical role during hematopoietic, especially megakaryopoietic, reconstitution following stem cell transplantation.

**Electronic supplementary material:**

The online version of this article (doi:10.1186/s13045-015-0152-2) contains supplementary material, which is available to authorized users.

## Background

Hematopoiesis is a tightly regulated process that leads to the well-balanced production of myeloid, erythroid, and lymphoid cells from a small number of highly proliferative hematopoietic stem and progenitor cells (HSCs and HPCs) [1]. During steady-state conditions, hematopoiesis is controlled by the coordinated action of a complex interplay of supporting growth factors and the signals they deliver through hematopoietic cytokine receptors expressed at the surface of HSPCs [2, 3]. These growth factors are produced in the bone marrow (BM) microenvironment or at multiple sites throughout the body from where they reach their target cells in the BM via the bloodstream [2, 4]. Broadly acting early cytokines, including interleukin (IL)-1, IL2, IL-3, IL-6, and IL-11, enhance the initial stages of hematopoietic development and can be distinguished from more late-acting hematopoietic differentiation-inducing cytokines like granulocyte colony-stimulating factor (G-CSF), macrophage colony-stimulating factor (M-CSF), and granulocyte-macrophage colony-stimulating factor (GM-CSF) [4]. Pattern recognition receptors like Toll-like receptors (especially TLRs 4, 7, and 9) recognize conserved microbial products derived from exogenous pathogens [5]. Inflammatory conditions like bacterial or viral infections increase the production and release of early- and late-acting hematopoietic cytokines, and these cytokines contribute to the rapid replenishment of consumed innate immune effector cells like granulocytes and macrophages [6]. As a result, early hematopoiesis in the BM is significantly skewed towards myeloid cell differentiation and output, leading to an increase of myeloid colony-forming progenitor cells as well as granulocytes in the circulation, a process that is described as emergency myelopoiesis [7, 8]. In response to gram-negative infections, emergency myelopoiesis is mediated by TLR4-expressing non-hematopoietic cells, which sense systemic lipopolysaccharide and by secreting myeloid cytokines such as G-CSF, induce an adequate myelopoietic response within the BM [9].

The involvement of different cytokines such as G-CSF, GM-CSF, or IL-6 in regulating hematopoiesis during steady states as well as during emergency situations has been shown: respective knockout mice have defects both in production and function of myelopoietic effector cells [10–12]. However, alternative pathways are likely to exist as mice with single or combined deficiencies for G-CSF, GM-CSF, and IL-6 or G-CSF and GM-CSF are still able to mount reactive myelopoietic responses during inflammatory conditions [10, 12–14].

The goal of this study was the identification of novel cytokines with yet unknown function in the hematopoietic system. We therefore analyzed the BM of lipopolysaccharide (LPS)- and vehicle-injected wild-type (WT) mice by gene expression microarray. Among the known candidates, we identified angiopoietin-like 4 (Angptl4) as a predominantly upregulated protein in the BM during inflammatory conditions. Angptl4 has a broad range of activities on hematopoiesis acting both on early hematopoietic progenitors as well as on immature CD61^+^CD41^low^-expressing megakaryocytes (MKs). Furthermore, Angptl4 is a potent stimulator of megakaryopoiesis after myelosuppressive therapy.

## Materials and methods

### RT-qPCR

For the isolation of RNA, tissue samples were homogenized in TRIzol reagent using a bead beater homogenizer (PEQLAB, Erlangen, Germany). All cell samples were lysed in TRIzol reagent, and RNA was purified according to the manufacturer’s instructions (Invitrogen, Carlsbad, USA). All RNA samples were subjected to DNAse I treatment. cDNA was synthesized using random hexamers primer and Superscript III reverse transcriptase according to the manufacturer’s instructions (Invitrogen, Carlsbad, CA, USA). For all expression experiments, 100 ng cDNA each was analyzed. Real-time PCR for Angptl4 and G-CSF mRNA expression in the murine BM, liver, spleen, lung, and bone marrow stromal cells, as well as PCR for friend leukemia integration 1 (Fli-1), signal transducer and activator of transcription 3 (STAT3), and nuclear factor erythroid-derived 2 (NF-E2) mRNA in MK cultures was performed using a sequence detector (7500 Fast Real-Time PCR System; Invitrogen) and TaqMan target mixes (Assay-on-Demand Gene expression reagents; Invitrogen).

### ELISA cytokine assay

Measurement of mouse G-CSF and Angptl4 was done in serum of LPS vs. control mice according to the manufacturer’s instructions (G-CSF, R&D Systems, Minneapolis, MN, USA; Angptl4, USCN Life Science, USA). BM plasma from the control and LPS-injected mice was prepared by flushing both femurs and tibia with 300 μl of cold PBS into Eppendorf-type centrifuge tubes. Cells/debris were removed by centrifugation at 3000 g for 10 min at 4 °C; BM plasma was stored at −20 °C. Control and LPS-stimulated bone marrow stromal cells (BMSCs) at passage 1 were grown to confluence in a T75 flask and kept for 48 h in 7 ml of Iscove’s Modified Dulbecco’s Medium (IMDM) (GIBCO; Life Technologies, Carlsbad, CA, USA) supplemented with 20 % FCS, 2 mM L-glutamine, 50 nM 2-mercaptoethanol (all reagents from Sigma-Aldrich, St. Louis, MO, USA), antibiotics (GIBCO; Life Technologies, Carlsbad, CA, USA), and with or without LPS (10 μg/ml) stimulation. Supernatants were harvested, cleared by centrifugation, and passed through a 0.45 μm filter. Culture supernatants were analyzed for G-CSF and Angptl4.

### Myeloid colony-forming assays

To assess colony-forming unit (CFU) stimulation of murine cytokines, freshly isolated mononuclear BM cells (3 × 10^4^) resuspended in IMDM and supplemented with 20 % FCS, 2 mM L-glutamine, 50 μM 2-mercaptoethanol, stem cell factor (SCF; 10 ng/ml), fms-related tyrosine kinase 3 (FLT3; 10 ng/ml), and thrombopoietin (TPO; 50 ng/ml) were mixed with methylcellulose (Methocult M3231, 2.6 %, StemCell Technologies, Vancouver, Canada) to yield a final concentration of 0.9 % methylcellulose. Additional factors were added in the following concentrations as indicated within the figure: IL-3 (20 ng/ml), GM-CSF (50 ng/ml), G-CSF (50 ng/ml), and Angptl4 (50 ng/ml). For estimation of CFU frequency after Angptl4 stimulation in vivo*,* 3 × 10^4^ cells were plated in methylcellulose mixed with IMDM (30 % FCS, 2 mM L-glutamine, 50 μM 2-mercaptoethanol) including the following factors: mIL-3 (10 ng/ml), hIL-6 (10 ng/ml), mSCF (10 ng/ml), mGM-CSF (10 ng/ml), mTPO (50 ng/ml), and huEPO (2 U/ml) (all R&D Systems, Minneapolis, MN, USA).

### Lethal irradiation and transplantation

Six- to ten-week-old female B6.SJL-PtprcaPep3b/BoyJ mice were lethally irradiated with 2 × 6.5 Gy in a 4-h interval and transplanted with 5 × 10^5^ BM mononuclear cells derived from syngeneic PBS, Angptl4, or non-injected donor mice. All mice were maintained at the animal facility of the university clinic in Aachen, Germany. All animal experiments were approved by the Federal Ministry for Nature, Environment and Consumers’ Protection of the state of North Rhine-Westphalia and were performed in accordance to the respective national, federal, and institutional regulations.

### LPS and Angptl4 injection

For microarray and mRNA analysis, the mice were injected once i.p. with 50 μg LPS (1:1 mixture of *Escherichia coli* K12 and *Salmonella minnesota*) and analyzed 8 h later. For detection of BM plasma and blood serum G-CSF and Angptl4 levels, the mice were injected twice i.p. with 50 μg LPS in a 48-h interval and analyzed 24 h later. Murine recombinant Angptl4 (250 μg/kg body weight in 100 μl PBS) was injected i.p. for five consecutive days, and the mice were analyzed 48 h later.

### In vitro generation of murine megakaryocytes

MKs were developed from lineage-depleted BM cells. Lin^+^ was depleted from mononuclear cells using a lineage cell depletion kit (Miltenyi, Bergisch Gladbach, Germany) according to the manufacturer’s instructions. Lin^−^ cells were seeded at 1 × 10^5^ cells per 1 ml in 48-well plates. Cells were cultured in IMDM containing bovine serum albumin, insulin, transferrin, SCF (25 ng/ml), and antibiotics. Where indicated, Angptl4 (30 ng/ml), TPO (30 ng/ml), or both were added. Cultures were performed at 37 °C in a fully humidified atmosphere of 5 % CO_2_. After 5 days of culture, cells were subjected to flow cytometry analysis using CD41 (eBioMWReg30) and CD61 (209.G3) antibodies, or smear preparations were prepared using cytospin and stained by the Wright-Giemsa method.

### Cell counting

Cells were counted using flow cytometry and Flow-Count fluorospheres (Beckman Coulter, Brea, CA, USA). After washing, harvested cells were resuspended in PBS containing 10 % FCS, 2 mM EDTA, and 7-aminoactinomycin D. Immediately prior to analysis, 50 μl of Flow-Count fluorospheres were added. Absolute cell counts were automatically determined using a Gallios FACS analyzer (Beckman Coulter, Brea, CA, USA). The system software calculated cell numbers using the following formula: cells per microliter = [(viable cells counted)/(fluorospheres counted)] × fluorospheres/microliter (see Additional file 1 for supplementary methods).

## Results

### Systemic inflammation regulates BM gene clusters associated with immune system process and positive regulation of cytokine production

**Fig. 1:**
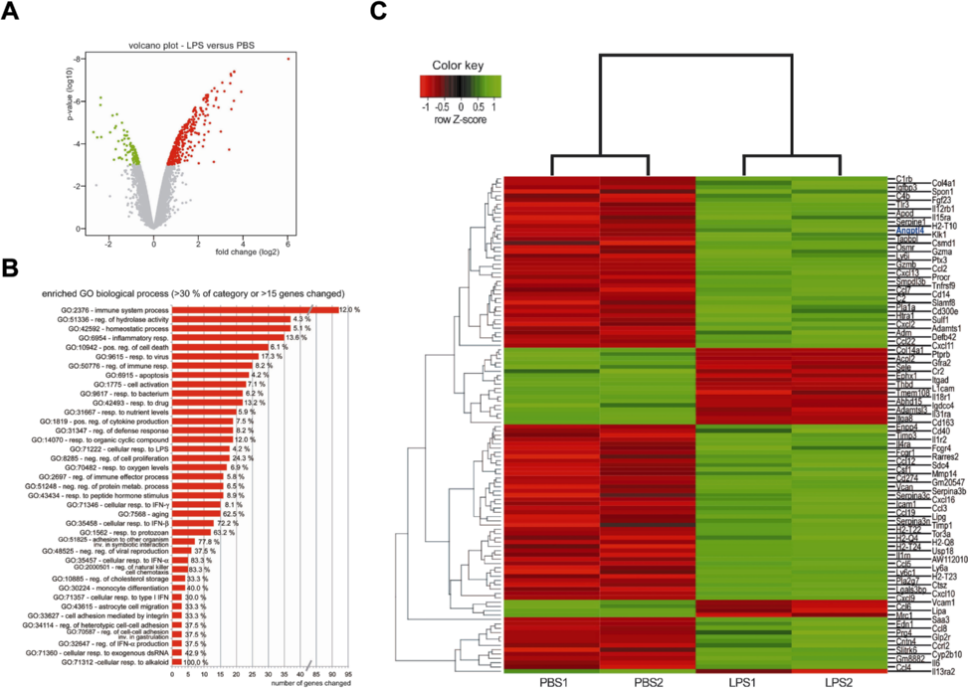
Systemic inflammation regulates BM gene clusters associated with immune system process and positive regulation of cytokine production. **a** Volcano plot for comparisons of BM cells isolated from LPS-treated (50 μg from 1:1 mixture of *E. coli* strain K12 and *S. minnesota* strain R595) and PBS-treated mice. Each gene is represented by a *dot* in the graph. The *x*-axis represents the log_2_ value of fold change, and the *y*-axis represents the *t*-statistic as log_10_ *p* value. *Colored dots* represent the genes that are regulated more or equal to 1.5 fold up (*red*, *n* = 326) or down (*green*, *n* = 86) with an adjusted *p* value not higher than 0.05. **b** GO analysis of regulated genes after LPS treatment. Enriched terms found related to regulated genes in biological processes (BP), operations, or sets of molecular events with a defined beginning and end and more than one distinct step. The *z*-score threshold of >1.96 resulted in 149 enriched GO terms for BP. Only enriched BP terms that include either 30 % or 15 differentially expressed genes are illustrated. **c** Heat map of differentially expressed genes predicted with the SignalP web server as cytokines or membrane proteins. After LPS treatment, 93 candidates are found to be upregulated and 23 to be downregulated. Genes are ordered in *rows* and samples in *columns*. The expression levels are coded as indicated in the color key. *Shades of green* and *red* refer to the differential expression levels as log_2_ fold values, as indicated in the color key

In order to find cytokines which are broadly enhancing myeloid cell regeneration, we analyzed the BM of the LPS- and vehicle-injected WT mice (i.p.; single injection) using oligonucleotide gene expression microarrays. Among 412 total genes, 326 were more than 1.5-fold and significantly (*p* ≤ 0.05) upregulated (Fig. 1a). To identify enriched gene ontology (GO) classes in any of the two differentially expressed gene sets, GO analysis was performed on GO-Elite using the gene ontology database [15, 16]. Biological categories such as immune system process, regulation of hydrolase activity, homeostatic process, and positive regulation of cytokine production were significantly enriched in the differentially expressed gene set, suggesting a generalized activation of the BM in response to inflammatory conditions (Fig. 1b). By focusing on the identification of genes encoding for potential cytokines or membrane proteins, we used signal peptide prediction algorithms to identify signal peptides which direct surface expression or secretion [17]. Among 116 predicted genes, 93 were upregulated and 23 were downregulated after LPS treatment (Fig. 1c). Among the known candidates, Angptl4 [18–20] was a predominantly upregulated gene in the BM of LPS-treated WT mice. Since previous studies had already implicated a potential role of angiopoietin-like family members affecting hematopoiesis [21, 22], we focused on the characterization of Angptl4 expression during inflammatory conditions and its effects on hematopoiesis in vitro and in vivo.

### Angptl4 is upregulated in the BM under inflammatory conditions

**Fig. 2:**
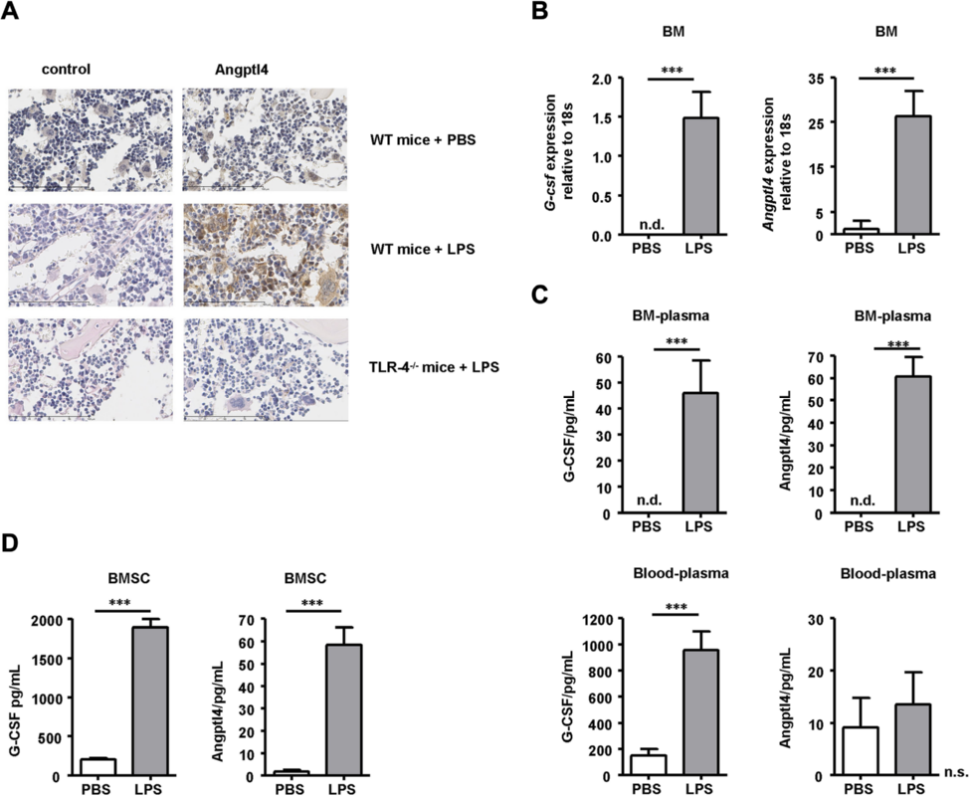
Angptl4 is upregulated in the BM of mice during inflammatory conditions. **a** Hematoxylin-eosin staining and Angptl4 expression in BM sections from WT and TLR-4^−/−^ mice at 72 h after double PBS or LPS (50 μg from 1:1 mixture of *E. coli* strain K12 and *S. minnesota* strain R595) injections. Sections were either labeled with anti-Angptl4 antibody (*right panel*) or isotype-matched antibodies (*left panel*). Original magnification ×200. **b** *G-csf* and *Angptl4* mRNA expression in the BM of PBS-treated (*white bars*) or LPS-treated (*gray bars*) WT mice. Expression levels are normalized against 18 s RNA. Mice were i.p. injected once with 50 μg LPS and analyzed 8 h later. Mean ± SEM of three different experiments each with a total of three PBS- and three LPS-injected mice/group are shown. **c** Blood and BM plasma G-CSF and Angptl4 protein levels in the PBS-treated (*white bars*) or LPS-treated (*gray bars*) WT mice, treated as in **a**. Mean ± SEM of two different experiments with a total of five PBS-treated and five LPS-treated mice are shown. **d** G-CSF and Angptl4 protein levels in supernatants at 48 h after single PBS (*white bars*) or LPS stimulation (10 μg from 1:1 mixture of *E. coli* strain K12 and *S. minnesota* strain R595, *gray bars*) of BMSC cultures. Mean ± SEM of supernatants from three experiments, each with different donor BMSCs, is shown. *n.d*. not detectable within the sensitivity of the assay, *n.s*. not significant within 35 cycles of amplification. Statistically significant differences are indicated (***p < 0.001)

To see if inflammatory signals translate into increased Angptl4 production at the protein level, we stained the BM sections of the WT and TLR-4^−/−^mice from the LPS-injected mice as well as the control injected WT mice with an antibody against Angptl4 (Fig. 2a). Strong Angptl4-positive cells were detected in the BM of the LPS-injected mice exclusively, including both non-hematopoietic stromal and endothelial cells as well as cells of hematopoietic origin as determined by morphological examination. We further evaluated Angptl4 upregulation during inflammatory conditions in comparison with G-CSF by qRT-PCR. We focused on G-CSF because during LPS-mediated inflammatory responses such as bacterial-induced inflammation or sepsis, G-CSF is heavily released albeit only detected on low levels in steady-state conditions [7, 8]. While *G*-*csf* mRNA was detectable in the total tissue extracts at low levels in steady-state spleen and lung which is in accordance with previous studies [23], this was initially not the case in the liver and BM (Fig. 2b and Additional file 2: Fig. S1A). However, at 8 h after i.p. LPS injection, *G-csf* mRNA expression was significantly upregulated in the BM, the primary sites of myelopoietic cell production, and in the liver as well as in the spleen and lung, sites of myelopoietic migration and activation (Additional file 2: Fig. S1A). *Angptl4* mRNA was detected at the baseline in the steady-state BM, lung, and spleen and upon inflammation was significantly and most extensively upregulated in the BM and lung and increased in the liver and spleen (Fig. 2b and Additional file 2: Fig. S1A). In line with *G-csf* and *Angptl4* mRNA induction, a significant increase of G-CSF and Angptl4 protein levels in BM plasma [24] was observed at 72 h after LPS injection in the WT mice, whereas in the vehicle-injected mice, G-CSF and Angptl4 protein levels were not detected (Fig. 2c). Upregulation of G-CSF in BM plasma after LPS injection was paralleled by high levels of G-CSF in blood plasma, whereas Angptl4 blood plasma levels were barely detectable and not different from the controls.

As different pathogenic signals evoke different cellular responses, we additionally analyzed mice with (*Streptococcus pneumoniae*) S.p.-induced experimental meningitis. After S.p. injection through the frontolateral skull, the mice have been shown to rapidly develop gram-positive bacteremia and a systemic inflammatory response [25], which is dependent on the activity of TLR-2, TLR-4, and TLR-9 [26–28]. We therefore analyzed the BM of the S.p.-injected mice for emergency myelopoiesis characteristic myeloid cell proliferation and differentiation [9, 24, 29]. As in the LPS-injected animals, mature myeloid cells (CD11b^+^Gr-1^high^) decreased upon S.p. injection, whereas the frequency of promyelocytes and myelocytes (CD11b^+^Gr-1^low^) were found to be increased (Additional file 2: Fig. S2A). Compared to the vehicle-injected animals, the S.p.-injected animals showed an increase of G-CSF and Angptl4 both in BM and in blood plasma, respectively (Additional file 2: Fig. S2B).

To identify the candidate producer cells of Angptl4 during inflammatory conditions, we purified mouse bone marrow stromal cells (BMSCs) from the BM by plastic adherence. Stromal cultures, developed from proliferating mesenchymal precursors, expressed stroma-associated surface markers and could be differentiated into adipocytes and osteoblasts (Additional file 2: Fig. S3A, B). Stimulation of murine BMSC with LPS induced the production of G-CSF and Angptl4 proteins, both of which were barely detectable at the baseline (Fig. 2d). We conclude that systemic inflammatory conditions induce *Angptl4* mRNA expression at different sites throughout the body including Angptl4 protein production and release in the BM, the primary site of hematopoiesis.

### Angptl4 stimulates the proliferation of myeloid progenitors in vitro and expands myeloid progenitors in vivo

**Fig. 3:**
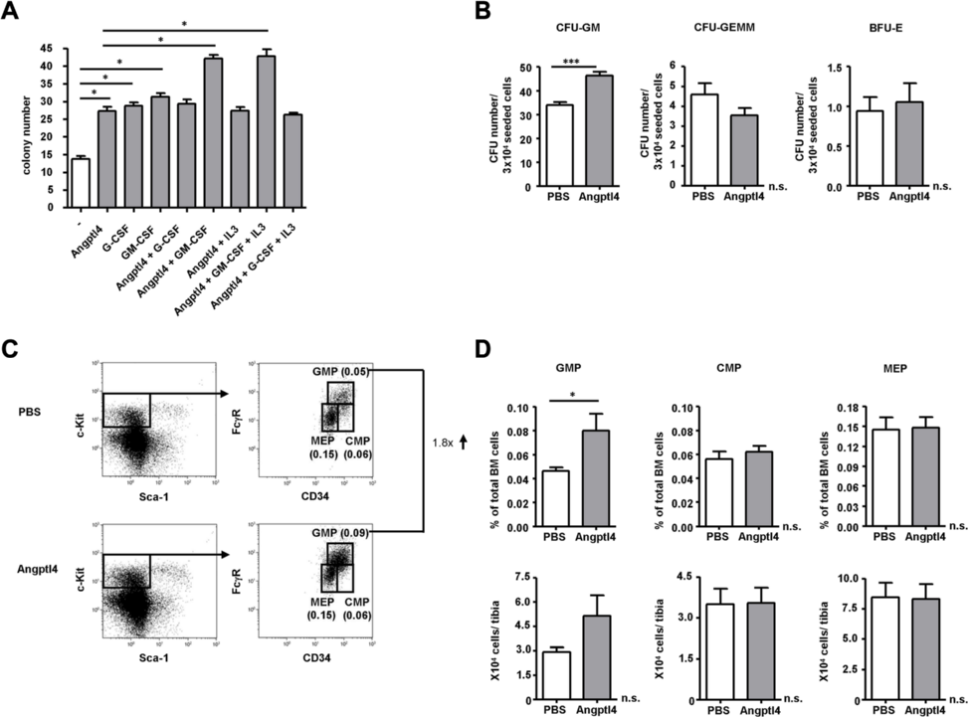
Recombinant Angptl4 stimulates the proliferation of myeloid CFUs in vitro and expands myeloid progenitors in vivo. **a** CFU activity of 3 × 10^4^ murine BM cells per well in the presence of SCF, TPO, and Flt3L. Addition of Angptl4, G-CSF, GM-CSF, and IL-3 as indicated. **b** CFU numbers per 3 × 10^4^ seeded BM cells of the PBS-treated (*white bars*) or Angptl4-treated (*gray bars*) WT mice in the presence of the following cytokines: IL-3, IL-6, SCF, GM-CSF, TPO, and EPO. Mice were daily injected with 250 μg/kg murine Angptl4 per kg body weight for five consecutive days. Mice were analyzed 48 h after the last injection. Mean ± SEM of two different experiments with a total of six PBS-injected and six Angptl4-injected mice are shown. **c** Representative FACS profiles of Lin^−^ BM cells from the PBS- and Angptl4-injected WT mice. Lin^−^ c-Kit^high^ Sca-1^−^ cells were subdivided into three subsets based on FcγRII/III and CD34 expression: CD34^+^FcγRII/III^−^ (CMP), CD34^+^FcγRII/III^+^ (granulocyte/monocyte progenitors; GMP), and CD34^−^FcγRII/III^−^ (MEP). Numbers indicate percentages of total BM cells and fold changes of CD34^+^FcγRII/III^+^ fractions in the PBS- vs. Angptl4-treated mice are shown. **d** GMP frequency as percentage of total cells, as well as total cell numbers in PBS- vs. Angptl-injected mice, treated as in **b**. Mean ± SEM of three different experiments with three PBS-injected and three Angptl4-injected mice/group are shown. Statistically significant differences are indicated (*p < 0.05, ***p < 0.001)

We first evaluated the in vitro effects of Angptl4 in myeloid CFU assays using murine BM cells (Fig. 3a). Treatment of BM cells with Angptl4 enhanced colony formation by approximately twofold, comparable to the stimulating activity of G-CSF and GM-CSF in this assay. Angptl4 did not further enhance the effects of IL-3 or G-CSF, alone or in combination. Treatment with Angptl4 and GM-CSF had additive effects on colony formation but this not further increased by the addition of IL-3. We therefore conclude that Angptl4 has a selective effect on colony formation towards a hematopoietic progenitor with responsiveness to GM-CSF but not to G-CSF. Next, we tested whether LPS-induced upregulation of Angptl4 in the BM correlates with enhanced progenitor cell growth and differentiation [30, 31] and examined colony formation of unfractionated BM with combined cytokines (SCF, TPO, IL-3, GM-CSF, IL-6, EPO) in vitro after Angptl4 treatment in vivo (Fig. 3b)*.* mrAngptl4 increased triglyceride levels in peripheral blood [32] and Angptl4 protein levels in the BM plasma of tail vein injected mice, suggesting that injected Angptl4 was functional and reached the BM cavity (Additional file 2: Fig. S4A). Colony numbers of granulocyte-macrophage progenitors (CFU-GM) were significantly increased after repeated Angptl4 injections, whereas the number of multilineage colony-forming unit-granulocyte, erythrocyte, monocyte/macrophage, megakaryocyte (CFU-GEMM) progenitors was reduced and the number of burst-forming unit-erythroid (BFU-E) colonies did not significantly differ between the Angptl4-treated and control animals. To determine whether the effects on colony formation recapitulates in cell-surface-defined hematopoietic progenitor subsets, we analyzed the impact of in vivo Angptl4 treatment on various progenitor populations by flow cytometry (Fig. 3c). While the number of granulocyte-macrophage progenitors (GMPs) was increased after administration of Angptl4, MK and erythrocyte lineage-restricted progenitors (MEPs) and common myeloid progenitors (CMPs) have not been affected (Fig. 3d). These data suggest that recombinant Angptl4 stimulates the proliferation of myeloid CFUs in vitro. In mice, repeated injections of Angptl4 increased BM progenitor cell frequency, and this was paralleled by a relative increase in phenotypically defined GMPs.

### In vivo treatment with Angptl4 results in elevated platelet counts in mice in steady state and after myelosuppressive therapy

**Fig. 4:**
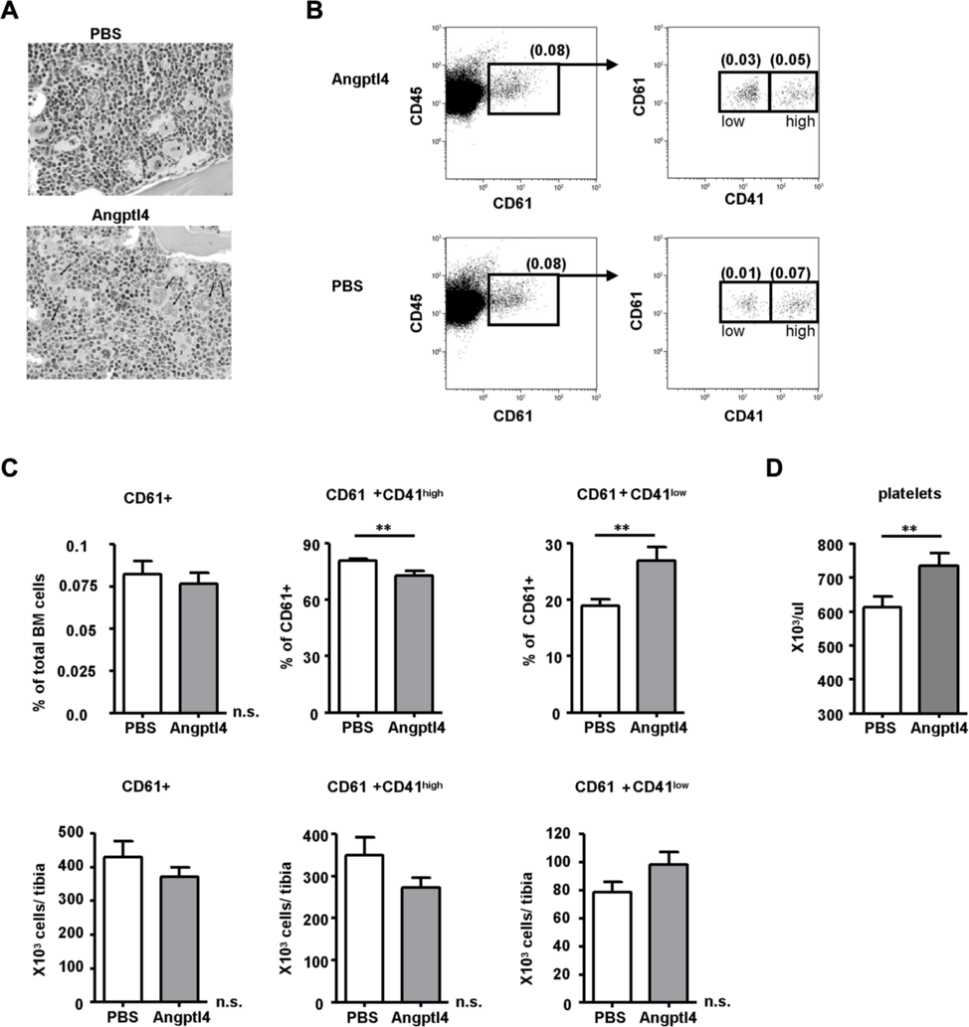
In vivo treatment with Angptl4 increases immature megakaryocytes and results in elevated platelet counts. **a** Hematoxylin-eosin staining of BM sections from PBS- or Angptl4-treated WT mice. Mice were daily injected with 250 μg/kg murine Angptl4 per kg body weight for five consecutive days and analyzed 48 h after the last injection. The symbol “*x*” indicates MKs in the BM of PBS- and rmAngptl-treated mice, and *arrows* indicate immature MKs in the BM of Angptl4-injected animals. One representative analysis from three independent experiments is shown. Magnification ×200. **b** Representative FACS profile of Lin^−^ BM cells from PBS- and Angptl4-injected WT mice. Lin^−^CD45^+^CD61^+^ cells were subdivided into three subsets based on CD61 and CD41 expression: CD61^+^, CD61^+^CD41^high^, and CD61^+^CD41^low^ cells. **c** Frequency and total cell numbers/tibia of CD61^+^, CD61^+^CD41^high^, and CD61^+^CD41^low^ cells. Mean ± SEM of three different experiments with three PBS-injected and three Angptl4-injected mice/group are shown. **d** PLT counts from PBS- or Angptl4-treated WT mice. Mean ± SEM of three different experiments with three PBS-injected and three Angptl4-injected mice/group are shown. Statistically significant differences are indicated (**p < 0.01)

As we did not recognize an effect on overall cellularity of the BM between the PBS- and Angptl4-treated animals, we went on to examine the effects of rmAngptl4 on more mature myeloid bone marrow cells by staining BM sections with hematoxylin and eosin. We identified local accumulations of dysplastic and immature MKs (Fig. 4a). Immature MKs recently have been described as a major population of MKs in murine BM and, based on low CD41 expression and acetylcholinesterase (AChE) activity, are defined as being Lin^−^/CD41^+^/CD45^+^/AChE^−^ [33]. In contrast, the more differentiated MKs are characterized by a substantial AchE activity and higher expression levels of CD41. By applying flow cytometry after surface staining on BM cells, we found within Lin^−^/CD45^+^ BM cells, CD61^+^CD41^low^ and CD61^+^CD41^high^-expressing MK subpopulations (Fig. 4b). Whereas the frequency of total MK numbers and CD61^+^CD41^high^-expressing MK subpopulations decreased after Angptl4 injection, the frequency of CD61^+^CD41^low^-expressing MKs increased significantly (Fig. 4b, c). The absolute numbers of MK subpopulation after Angptl4 injection showed a similar trend but did not reach significance with the number of experiments performed.

**Fig. 5:**
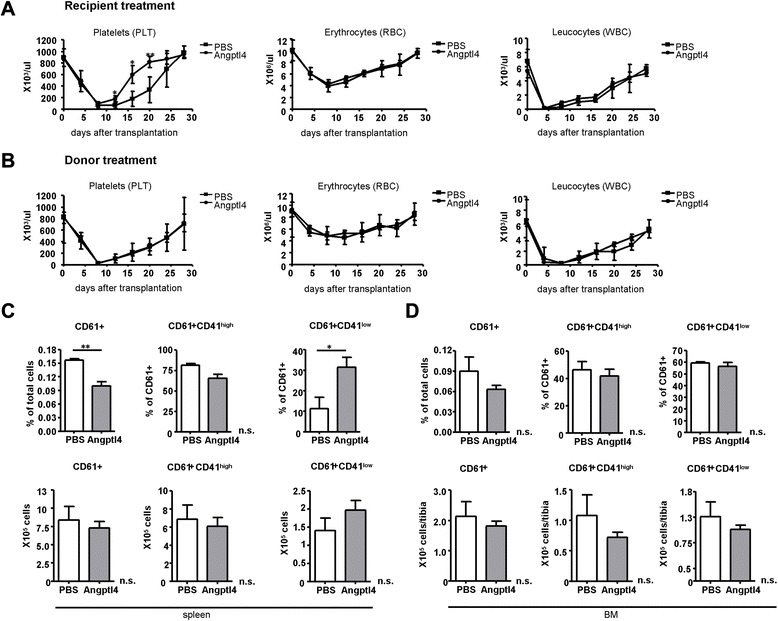
Angptl4 accelerates the reconstitution of megakaryopoiesis after lethal irradiation and transplantation. **a** Reconstitution of PLTs, erythrocytes (RBCs), and leucocytes (WBCs) after lethal irradiation (2 × 6.5 Gy) and transplantation of 1 × 10^5^ BM cells. After transplantation, mice were daily injected with PBS (shown in *squares*) or murine Angptl4 (shown in *circles*; 250 μg/kg body weight) for five consecutive days. Peripheral blood of transplanted mice was collected every 3 days for a time course of 30 days, and PLT, RBC, and WBC counts were determined using an animal blood counter. Each time point represents the mean of two different experiments with a total of ten mice/group. **b** PLT, RBC, and WBC counts after lethal irradiation (2 × 6.5 Gy) and transplantation of 1 × 10^5^ BM cells from Angptl4 (*circles*) or PBS (*squares*) pre-treated donor mice. Donor mice were daily injected with PBS or Angptl4, as described in **a** and sacrificed 48 h after the last injection. After transplantation, peripheral blood was collected and analyzed as described in **a**. Each time point represents the mean of two different experiments with a total of ten mice/group. **c** Frequency and total cell numbers of CD61^+^, CD61^+^CD41^high^, and CD61^+^CD41^low^ cells in the spleen at day 30 after transplantation. Recipient mice were treated with Angptl4 after transplantation, as described in **a**. Mean ± SEM of three different experiments with three PBS-injected (*white bars*) and three Angptl4-injected (*gray bars*) mice/group are shown. **d** Frequency and total cell numbers of CD61^+^, CD61^+^CD41^high^, and CD61^+^CD41^low^ cells in the BM at day 30 after transplantation. Recipient mice were treated with Angptl4 after transplantation, as described in **a**. Mean ± SEM of three different experiments with three PBS-injected (*white bars*) and three Angptl4-injected (*gray bars*) mice/group are shown. (*p < 0.05, **p < 0.01)

Concomitantly, platelet (PLT) numbers were increased in the Angptl4-injected mice (Fig. 4d) suggesting an effect of Angptl4 specifically on the frequency of CD61^+^CD41^low^-expressing MKs resulting in an acceleration of differentiation during megakaryopoiesis. Acceleration of megakaryopoiesis is believed to be the most effective way to achieve a rapid recovery of PLT numbers after cancer chemotherapy or stem cell transplantation [34, 35], and cytokines such as IL-3, IL-6, IL-11, and TPO have been shown to increase MK and PLT numbers after transplantation of myelosuppressed animals [36–38]. We therefore assessed the effect of Angptl4 injection on PLT reconstitution after lethal irradiation and transplantation of BM cells into recipient mice. Repetitive injections of recombinant Angptl4 for five consecutive days resulted in a significantly accelerated reconstitution of PLTs starting at day 8 after transplantation (Fig. 5a). The 50 % pre-treatment PLT count was reached on day 14 in the Angptl4-treated animals, as compared to day 21 for transplanted controls receiving no Angptl4. In contrast, we were unable to detect differences in the reconstitution levels of erythrocytes and leucocytes between the Angptl4- and PBS-injected animals at any time point examined further supporting the specific effect of Angptl4 on megakaryopoiesis (Fig. 5a). The accelerated post-transplant recovery of PLTs after Angptl4 injection may be due to the expansion of the number of megakaryocyte colony-forming units (CFU-Meg) or may be related to effects on more mature megakaryocytic progenitor cells. To distinguish between these possibilities, we assessed the effect on PLT reconstitution after transplantation of in vivo Angptl4-modified BM cells. The donor mice were treated with Angptl4 for five consecutive days, and their BM was used for transplantation at day 7. The control animals were transplanted with BM cells from the PBS pre-treated donor mice (Fig. 5b). As compared to the control animals, in vivo modification of donor BM with Angptl4 did not result in an accelerated reconstitution of PLT numbers. This suggests that an effect of Angptl4 at the level of CFU-Meg seems very unlikely. Instead, we believe that Angptl4 specifically accelerates the differentiation process of MKs. In line with this, we found significantly increased relative cell numbers of CD61^+^CD41^low^ cells in the spleen of the post-transplanted Angptl4-treated mice (Fig. 5c) and this was recapitulated with a non-significant trend in absolute cell numbers. It has previously been shown that after lethal irradiation and transplantation, donor-derived megakaryopoiesis occurs in the spleen at higher densities than in the BM [39]. In line with this observation, a post-transplant effect of Angptl4 on CD61^+^CD41^low^- and CD61^+^CD41^high^-expressing MKs within the BM was not detected (Fig. 5d).

### Angptl4 increases the number of immature megakaryocytes in vitro

**Fig. 6:**
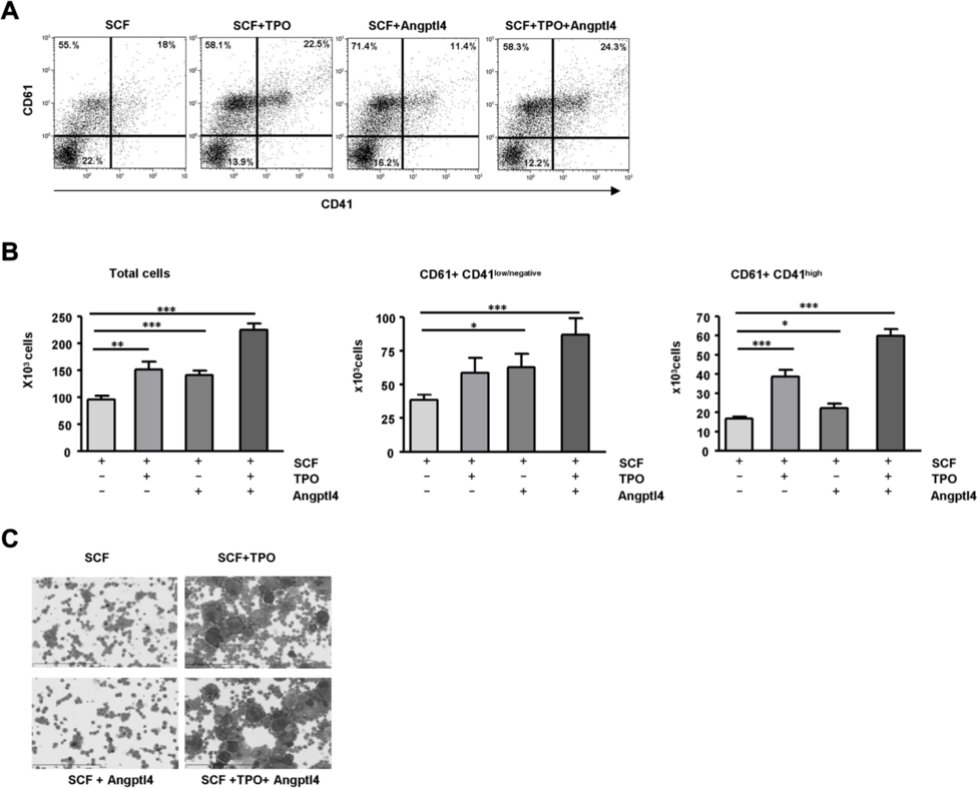
Angptl4 increases the number of CD61 + CD41^low/negative^ MKs in vitro*.* **a** Representative FACS profile of CD61^+^CD41^+^, CD61^+^CD41^low/negative^, and CD61^−^CD41^−^ cells in SCF-, SCF + TPO-, SCF + Angptl4-, and SCF + TPO + Angptl4-supplemented cultures at day 5 after initiating cultures from 1 × 10^5^ Lin^−^ BM cells. **b** Total cells developed and total number of CD61^+^CD41^low/negative^ and CD61^+^CD41^+^ cells in cytokine-supplemented cultures as described in **a**. Mean ± SEM of three different experiments with a total of 12 SCF-; 12 SCF and TPO-; 12 SCF and Angptl4-; and 12 SCF, Anptl4, and TPO-supplemented cultures are shown. Statistically significant differences are indicated (*p < 0.05, **p < 0.01, ***p < 0.001). **c** Representative cytospin preparation and Wright-Giemsa staining of MK cultures as described in **a**. Original magnification ×400

To see if Angptl4 has impact on megakaryopoiesis in vitro, we generated MKs by a serum-free liquid culture system of enriched BM hematopoietic stem and progenitor cells in the presence of SCF and the addition of TPO, Angptl4, or combined TPO and Angptl4. Based on surface staining and flow cytometry, we distinguished CD61^+^CD41^low/negative^ megakaryocytic lineage cells from CD61^+^CD41^high^ megakaryocytic lineage cells (Fig. 6a). After 5 days of culture, the addition of Angptl4 or TPO increased total cell numbers as compared to culture with SCF alone, with the highest cell numbers recovered from combined Angptl4 and TPO cultures. CD61^+^CD41^low/negative^ as well as CD61^+^CD41^high^ MK lineage cell numbers were increased in both Angptl4-supplemented cultures above levels obtained with SCF only (Fig. 6b). The effect on the development of CD61^+^CD41^low/negative^ cells was comparable between Angptl4- or TPO-supplemented cultures, whereas the Angptl4 effect on CD61^+^CD41^high^ cells was much lower (Fig. 6b). In addition, additive effects in Angptl4- and TPO-combined cultures could be observed both on the development of CD61^+^CD41^high^ MKs as well as on the development of CD61^+^CD41^low/negative^ cells (Fig. 6b). This suggests that Angptl4 and TPO expand immature CD61^+^CD41^low/negative^ cells and that, in Angptl4 and TPO combination cultures, the expanded CD61^+^CD41^low/negative^ cells are responsive to differentiation induced by TPO. In line with previous reports [40], we found terminally differentiated MKs including cytoplasmic maturation and expansion in TPO-supplemented cultures only (Fig. 6c). As shown by cytospin preparation and Wright-Giemsa staining, TPO-supplemented cultures developed large, smooth MKs, whereas in the absence of TPO, small and medium size cells could be found. This data suggest that Angptl4 increases immature megakaryopoietic progenitor cell numbers in vitro but that it has no effect on terminal differentiation of MKs including cytoplasmic maturation.

### Angptl4 induces de novo STAT3 expression in CD61^+^CD41^low/negative^ megakaryocytes in vitro

MK maturation is governed by the activity of a group of transcription factors, including GATA-1, ETS family members, NF-E2, and STAT3 [41, 42]. To investigate the relative mRNA expression levels of transcription factors associated with different stages of MK differentiation in response to Angptl4, we chose the ETS family member Fli-1 [43–45], STAT3 [46, 47], NF-E2 [48], and the GATA binding protein GATA-1. NF-E2 expression is associated with late cytoplasmic maturation stages of MK development [49]; Fli-1 and STAT3 are believed to be expressed on MK precursors through the promegakaryoblast stage with additional expression of STAT3 in mature megakaryocytes and platelets [46, 47, 50, 51]. GATA-1 has been shown to promote MK differentiation and to restrain abnormal immature MK expansion [52].

**Fig. 7:**
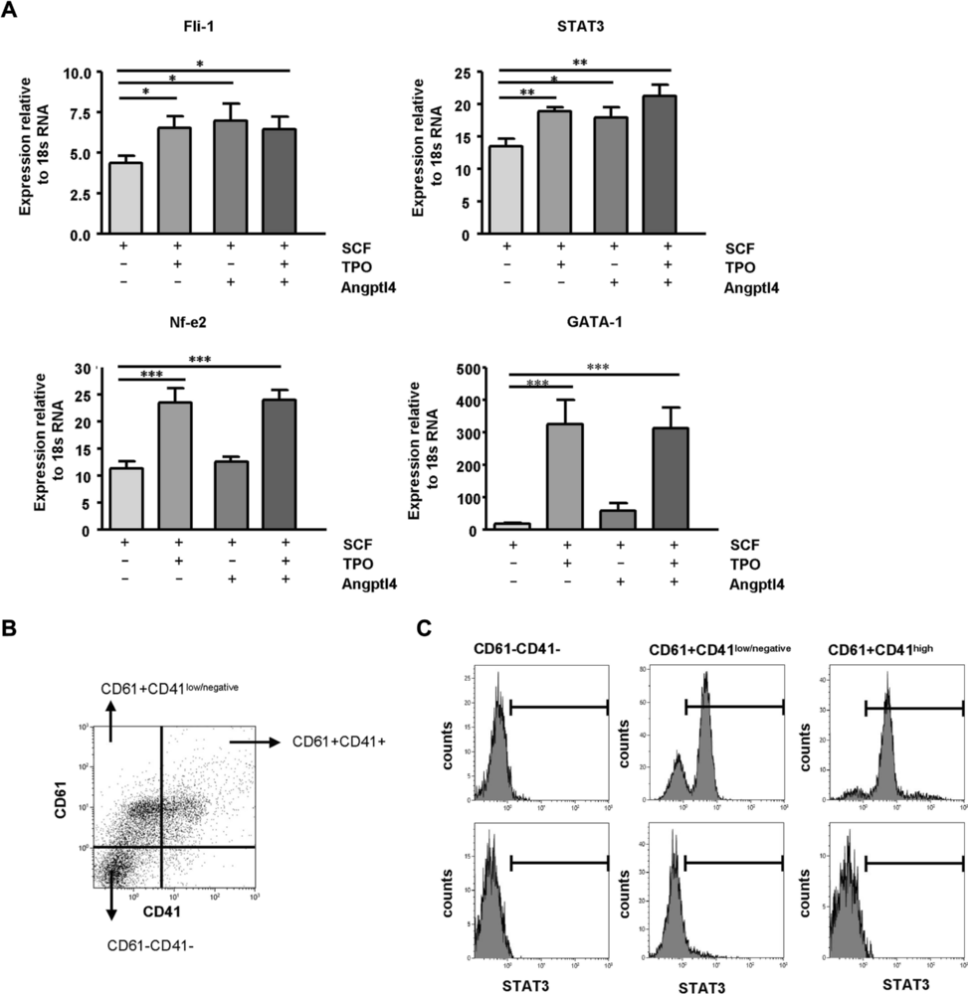
Angptl4 induces de novo STAT3 expression in CD61^+^CD41^low/negative^ MKs in vitro*.* **a** mRNA expression of the transcription factors Fli-1, STAT3, NF-E2, and GATA-1 in SCF-, SCF + TPO-, SCF + Angptl4-, and SCF + TPO + Angptl4-supplemented cultures at day 5 after initiating cultures from 1 × 10^5^ Lin^−^ BM cells. Mean ± SEM of three different experiments with a total of 12 SCF-; 12 SCF and TPO-; 12 SCF and Angptl4-; and 12 SCF, Angptl4, and TPO-supplemented cultures are shown. Statistically significant differences are indicated (*p < 0.05, **p < 0.01, ***p < 0.001). **b**, **c** Representative FACS profile and gating strategy for the detection of STAT3 expression of CD61^−^CD41^−^, CD61^+^CD41^low/negative^, and CD61^+^CD41^high^ cells in cytokine-supplemented cultures at day 5 after initiating cultures from 1 × 10^5^ Lin^−^ BM cells. **c** *Upper panel*: initiating cells are purified from STAT3-YFP knockin mice; *lower panel*: initiating cells stem from WT mice. Statistically significant differences are indicated (*p < 0.05, **p < 0.01, ***p < 0.001)

Fli-1 and STAT3 mRNA expression levels were both induced by recombinant Angptl4 as well as TPO, confirming an involvement of Angptl4 during early developmental stages of MK differentiation (Fig. 7a). In contrast, an increase in NF-E2 mRNA expression could only be found in TPO-supplemented cultures which is in line with a lack of effect of Angptl4 in late cytoplasmic maturation stages of MK development. Similarly, when we analyzed GATA-1 mRNA, GATA-1 expression increased to very high levels in the presence of TPO (Fig. 7a TPO). We additionally analyzed GATA-1 and STAT3 in Angptl4-supplemented “immature” cultures by confocal microscopy and found GATA-1 in contrast to STAT3 barely detectable (Additional file 2: Fig. S5D; immature/Angptl4). In line with mRNA expression data, GATA-1 detection increased in mature MKs.

Therefore, the expression levels for Fli-1, STAT3, NF-E2, and GATA-1 mRNA reflect whether the culture contains mainly immature or mature MKs.

It was shown that GATA-1 could bind and inhibit the activity of STAT3 in MKs [52] and that the transgenic expression of dominant negative STAT3 causes a significant delay in PLT recovery after myelosuppression, suggesting that STAT3 is required for normal regulation of megakaryopoiesis [47]. We therefore focused on STAT3 to determine the effects of Angptl4 on this transcription factor in more detail. To confirm that upregulation of STAT3 mRNA levels by Angptl4 faithfully reflects increased STAT3 protein levels and to identify STAT3 expression in megakaryocytic and non-megakaryocytic lineage cells in culture, we made use of a mouse model with STAT3-yellow fluorescent protein (YFP) knocked into the endogenous STAT3 locus. The mice bearing the STAT3-YFP knockin allele develop normally are fertile and display normal development of different myeloid lineages, such as monocytes, granulocytes, and MKs (data not shown). By measuring YFP, we assessed the expression of STAT3 in MK cultures at day 5 after initiating from Lin^−^/STAT3-YFP BM cells. In all cytokine-supplemented cultures, STAT3^+^ cells developed with relative number varying between 15–60 % (Additional file 2: Fig. S4A, B and data not shown) and CD61^+^CD41^low/negative^ megakaryocytic lineage cells as well as CD61^+^CD41^high^ megakaryocytic lineage cells could be identified. In non-megakaryocytic lineage cells defined by the lack of CD61 and CD41 surface staining, STAT3^+^ cells were not detected, suggesting a restriction of STAT3 expression to developing megakaryocytic lineage cells in these cultures (Fig. 7b, c). In line with that, STAT3 expression could be clearly detected in CD61^+^CD41^low/negative^ as well as CD61^+^CD41^high^-expressing cells (Fig. 7c). In CD61^+^CD41^low/negative^ cells, Angptl4 increased and at the same time TPO decreased the fraction of STAT3^+^ cells. In CD61^+^CD41^high^ cells, TPO increased the percentage of STAT3^+^ cells whereas Angptl4 had no additional effect. The number of STAT3^+^ cells therefore reflects STAT3 mRNA levels and further supports the impact of Angptl4 action towards immature megakaryocytic lineage cells.

## Discussion

In this study, we identified Angptl4 as an upregulated protein during inflammatory conditions in the BM of mice and determined the effects of recombinant Angptl4 on early and late stages of hematopoieisis in vitro and in vivo. Angptl4 is a secreted glycoprotein with a physiological role in lipid metabolism and a predominant expression in adipose tissue and liver [20, 53, 54]. Angptl4 inhibits the activity of lipoprotein lipase and thereby promotes an increase in circulating triglyceride levels [32, 55, 56]. Further involvement of Angptl4 has been shown to occur in energy homeostasis, wound repair, tumorigenesis, angiogenesis, and redox regulation [53]. As a member of the superfamily of Angptl proteins (including Angptl 1–7), Angptl4 shares sequence homology with angiopoietins and consists of a secretory signal peptide, an N-terminal coiled-coil domain, and a C-terminal fibrinogen-like domain [53]. In close correlation to our data, upregulation of Angptl4 expression has been shown during acute phase responses in the LPS-treated mice in the liver, heart, muscle, and adipose tissue [57]. It was suggested that elevated levels of Angptl4 could contribute to hypertriglyceridemia, which is associated with inflammation; however, a direct effect of Angptl4 on inflammatory pathways has not been demonstrated so far. We here connect inflammation-induced upregulation of Angptl4 with the first hallmark of emergency myelopoiesis: the increase of myeloid progenitors in the BM. However, as LPS induces the production of a variety of different cytokines and multiple cytokines display redundant functions on hematopoiesis, the definitive role of Angptl4 in mediating the effects of LPS on the BM has to be demonstrated by combining multiple cytokine knockouts.

Members of the angiopoietin-like family of proteins, including Angptl4, have been shown to bind to paired immunoglobulin-like receptors, which supports *ex vivo* expansion of HSCs [21]. Remarkably, Angptl4 did not effectively stimulate expansion of HSCs. When transplanted into conditioned mice, Angptl4 expanded HSCs and supported engraftment for 4 weeks only; however, such effects were not detected 3 or 6 months after transplantation [21]. These findings imply that Angptl4 might have effects on committed progenitor cells rather than on immature self-renewing HSCs. Indeed, our results show that Angptl4 can regulate myeloid cell proliferation at the level of HPCs, and Angptl4 therefore should be added to the growing list of early-acting cytokines such as IL-3 and IL-6. Interestingly, Angptl4, G-CSF, and GM-CSF were equally active in enhancing agents for murine BM colony formation in the presence of SCF, TPO, and Flt3L in vitro. However, when combined, Angptl4 had additive effects with GM-CSF but not with G-CSF to increase colony formation. The mechanism for this action is not known but may involve an increased sensitivity of CFUs to Angptl4 or GM-CSF through receptor upregulation or maturation of progenitors.

In contrast to the action of G-CSF, Angptl4 seems to have more local and limited effects. As the receptor of G-CSF is expressed throughout the granulocytic lineage from granulocytic precursors to mature neutrophils, its increase in the blood has an immediate impact on granulopoiesis [4]. A massive increase in G-CSF upon LPS stimulation is therefore, as a single cytokine, sufficient to translate into leukocytosis and neutrophilia, which is observed during emergency myelopoiesis [9]. Angptl4 has been shown to be regulated by hypoxia and chronic inflammatory responses. Angptl4 functions as a matricellular protein and by inhibiting lipoprotein lipase (LPL), Angptl4 increases serum trigylceride levels [57]. Although Angplt 4 is seen as a physiological mediator of intracellular adipose tissue lipolysis, serum Angptl4 levels do not always correlate with plasma triglyceride levels [58]. It has already been demonstrated that hematopoietic stem cells (HSCs) express PIRB, and PIRB therefore may function as sensor of inflammation through binding to the inflammatory Angptl4 and protecting HSCs from excessive activation and exhaustion [59].

To see if Angptl4 has any in vivo action, we injected mrAngptl4 into the WT mice for five consecutive days. At the level of progenitor cells, the results obtained showed that in vivo application stimulates the same types of HPCs as are stimulated in vitro by mrAngptl4. However, the effects seen after mrAngptl4 injection were relatively moderate and did not result in a significant increase of white blood cell counts. In addition, we directly assessed the effects on myeloid cells after mrAngptl4 in vivo injection as compared to LPS injection. Whereas LPS treatment clearly induced an increase in promyelocytes and myelocytes in vivo, repeated Angptl4 injection had only a marginal effect on the induction of metamyelocytes and band forms (data not shown). We therefore conclude that Angptl4 might be involved in the fine-tuning of proliferation and differentiation of leukocytes at the level of their progenitors.

In vivo mrAngptl4 applications further lead to an increase of peripheral blood PLTs and an increase of immature MKs in the BM, whereas phenotypically defined MEPs were not affected. In addition, when applied after lethal irradiation and transplantation, mrAngptl4 treatment resulted in a significantly accelerated recovery of PLTs, whereas the transplantation of mrAngptl4 in the in vivo pre-treated BM cells did not, suggesting that Angptl4 exerts its effects mainly on immature MKs downstream of CFU-Meg. In line with that, cytokines, which have been shown to be capable of modifying murine BM cells to accelerate PLT reconstitution after transplantation, such as IL-6 and TPO [36–38], also have been shown to stimulate and/or expand CFU-Meg formation in murine cultures and in vivo*.* Therefore, it is tempting to speculate that Angptl4 induces platelet production through the interaction with monopotent megakaryocyte-committed progenitors (MKPs) [60]. Common myeloid progenitors (CMP) and megakaryocyte-erythrocyte progenitors (MEPs) can differentiate into MKPs after 72 h in stromal cultures, indicating that MKPs are downstream of these two progenitors. MKPs are cytokine responsive progenitor cells, which do not display self-renewal activity and therefore give rise to platelets for approximately 3 weeks [60]. When we analyzed c-kit^+^Sca-1^−^CD150^+^ CD41^+^Lin^−^ MKPs, about 50 % of the cells were positive for PIRB (Additional file 2: Fig. S4B). These findings therefore suggest that the increased production of platelets can be mediated by direct interactions between secreted Angptl4 and its receptor, expressed on early MK-committed progenitor cells.

In contrast to MEPs, MKPs do not display any spleen colony-forming activity [61]. The increase of megakaryopoiesis in the spleen after transplantation therefore depends on the number of MEPs, trafficking from the recovering bone marrow to the spleen. The fact that after transplantation, Angplt4 increases relative cell numbers of CD61^+^CD41^low^ cells in the spleen but not in the BM (Fig. 5) therefore most likely reflects the post-transplant distribution of developing MKPs. In addition, different effects of systemic available Angptl4 on the BM and spleen cannot be excluded at this point.

After LPS injection, the TLR4−/− mice were shown to have a decreased circulating and reticulated platelet count compared to the WT mice. In the WT mice, platelets increased 1 week after a single-dose LPS injection, and this is beyond the time it takes for the circulating platelet pool to turnover in the mice [62]. As platelets have no nuclei, these findings therefore suggest a contribution of TLRs (direct or cytokine mediated) for genomic regulation of platelet production at the level of MKs. In support for this notion are findings which demonstrate that inflammatory processes can increase MK maturation and protein content through TLR2, which may affect platelet function and thrombosis. In line with this, our findings suggest that the increased production of platelets during inflammation can be mediated by direct interactions between secreted Angptl4 and its receptor, expressed on early MK-committed progenitor cells.

The fact that after Angptl4 injection, an increase in immature megakaryocytes and a lack of increase in mature megakaryocytes lead to the production of platelets seems to be counterintuitive at first sight. The in vitro differentiation of CD61^+^CD41^low^ MKs into platelet-producing CD61^+^CD41^high^ MKs has been demonstrated, and by following the development of immature CD61^+^CD41^low^, mature CD61^+^CD41^high^, and platelets for several days, their in vivo dynamics after IL-11 injection suggested that the subsequent increase in CD61^+^CD41^high^ MKs and platelets was due to de novo maturation from CD61^+^CD41^low^ MKs [33]. Conversely, we did not detect any increase in CD61^+^CD41^high^-expressing MKs after Angptl4 injection.

The accumulation of immature MKs could be either due to an inhibition of the maturation of MKs by Angptl4, the lack of local cytokines which promote terminal differentiation of MKs, or alternatively a proapoptotic effect on mature MKs. For instance, a large number of mature megakaryocytes that appear to undergo cell death as a result of IL-6 administration have been noted previously [63], and at the same time, IL-6 has a platelet-enhancing effect and promotes megakaryocyte maturation and colony formation [64, 65]. In addition, both intrinsic and extrinsic apoptosis pathways have been described to be implicated in thrombopoiesis. The effects reported after Angptl4 injection therefore may be directly or indirectly related to IL-6 and be part of a homeostatic control mechanism, counterbalancing megakaryocyte hyperplasia. As MK cells have previously been shown to produce IL-6 [66], we measured IL-6 levels in cytokine-supplemented cultures. After 9 days of culture, the addition of Angptl4 or TPO increased IL-6 levels as compared to culture with SCF alone, with the highest level recovered from combined Angptl4 and TPO cultures (Additional file 2: Fig. S4C).

Furthermore, it has been shown that platelets express PIRB as well as the ortholog of human leukocyte immunoglobulin-like receptor B2 (LILRB2), and Angptl2 has been shown to inhibit platelet activation partially through the PIRB pathway [67].

In any case, large numbers of CD61^+^CD41^low^ MKs, induced by Angptl4, lead to their differentiation into CD61^+^CD41^high^ MKs and an increase in rapid platelet recovery in vivo*.* This conclusion is strongly supported by our in vitro findings: as shown in Fig. 6b, immature CD61^+^CD41^low/negative^ MK numbers are increased in Angptl4-supplemented cultures and this leads to an additive increase in mature CD61^+^CD41^high^ MKs in Angptl4- and TPO-combined cultures.

Angptl4 induces STAT3 protein expression in CD61^+^CD41^low/negative^ megakaryocytic lineage cells, and STAT3 has been suggested to be important for effective expansion of megakaryocytic progenitor cells in the early stage of megakaryopoiesis, as shown by the delayed recovery of PLTs after myeloablation in mice carrying a dominant negative version of STAT3 [47]. We here establish a functional link between Angptl4 and STAT3 and propose a model in which Angptl4 through regulation of STAT3 expression expands immature MKs, which in the setting of autologous stem cell transplantation represents a potential approach to accelerate the reconstitution of megakaryopoiesis.

## Conclusion

Based on the fact that single or combined deficiencies of known hematopoiesis supporting cytokines do not abrogate emergency hematopoiesis, we looked for inflammation-induced cytokines with a yet unknown function in the hematopoietic system. By identifying Angptl4, we provide a new player with effects both on early hematopoietic progenitors as well as on platelet production. We envision further studies aiming at a possible role of Angptl4 as a predictor of platelet engraftment after autologous stem cell transplantation or its use in the acceleration of engraftment in clinical transplantation settings.

## Additional files

Additional file 1:
**Supplementary Methods.**
Additional file 2:
**Supplementary Figure.**
