# Supplementary material for: Angptl4 is upregulated under inflammatory conditions in the bone marrow of mice, expands myeloid progenitors, and accelerates reconstitution of platelets after myelosuppressive therapy
Source: J Hematol Oncol. 2015 Jun 9;8:64. doi: 10.1186/s13045-015-0152-2 (PMC4460974; doi:10.1186/s13045-015-0152-2)
Supplement: Additional file 1: — Supplementary Methods. [file 13045_2015_152_MOESM1_ESM.doc]

**SUPPLEMENTAL INFORMATION TO:**

Angptl4 is upregulated during inflammatory conditions in mice, expands myeloid progenitors and accelerates reconstitution of platelets after myelosuppressive therapy.

**MATERIAL AND METHODS**

**Gene expression profiling by microarray analysis**

Gene expression in BM cells isolated from PBS- and LPS-treated B6.SJL-PtprcaPep3b/BoyJ mice were analyzed by the GeneChip MouseGene 1.0 ST (Affymetrix, Santa Clara, CA, USA) in independent duplicates. Total RNA was extracted with TRIzol and quantified (Nanodrop, Peqlab, Erlangen, Germany). RNA quality was assessed using RNA 6000 NanoAssay with the 2100 Bioanalyzer (Agilent, Santa Clara, CA, USA). Probes for the GeneChip Mouse Gene 1.0 ST arrays were prepared and hybridized to the arrays according to the Ambion whole-transcript expression and the Affymetrix whole-transcript terminal labelling and control kit manuals. Briefly, for each sample, 300 ng of total RNA was reversetranscribed into cDNA using a random hexamer oligonucleotide tagged with a T7 promoter sequence. After second strand synthesis, double-stranded cDNA was used as a template for amplification with T7 RNA polymerase to obtain antisense cRNA. Random hexamers were then used to reverse-transcribe the cRNA into single-stranded sense strand cDNA. The cDNA was then fragmented by uracil DNA glycosylase and apurinic/apyrimidic endonuclease 1. Fragment size was checked using the Agilent 2100 Bioanalyzer (fragment size between 50– 200 bp). Fragmented sense cDNA was biotin-labelled with TdT and probes were hybridised to the GeneChip MouseGene 1.0 ST arrays at 45°C for 16 hours. Hybridised arrays were then washed and stained on a Fluidics Station 450 and scanned on a GeneChip® Scanner 3000 7G (both Affymetrix, Santa Clara, CA, USA). The image data were analysed with GeneChip Command Console software (Affymetrix, Santa Clara, CA, USA). To assess differential expression, CEL files were normalized by robust multiarray average to facilitate comparisons across arrays using the AltAnalyze 2.0.8 beta software suite (Gladstone Institutes, San Francisco, CA, USA) [1]. Default analysis options were used, which included analysis of gene expression via an empirical Bayes moderated t-test with a Benjamini-Hochberg-adjusted p-value [2]. Transcripts with a fold change of 1.5 or more and an adjusted p-value of less than 0.05 between the LPS- and PBStreated groups were considered as being differentially expressed. Cluster analyses were performed using the k-means clustering algorithm. To identify enriched gene ontology (GO) classes (z-score threshold: 1.96) in any of the two differentially expressed gene sets, GO analysis was performed on GO-Elite (Gladstone Institutes, San Francisco, CA, USA) [3]. Array data have been deposited in the Gene Expression Omnibus database (http://www.ncbi.nlm.nih.gov/geo/) under accession number GSE55161.

**Isolation of murine BMSC.**

Murine BMSCs were isolated by plastic adherence from bone marrow. After Ficoll gradient purification, cells were plated in FCS pre-coated T75 flasks in Iscove’s Modified Dulbecco’s Medium (IMDM) with 15% FCS (HyClone defined, Logan, USA), penicillin (50 U/ml; GIBCO, Carlsbad, USA), streptomycin (0.05 mg/ml; GIBCO, Carlsbad, USA) and dexamethasone (10-8 M; Sigma-Aldrich, St. Louis, USA). After overnight incubation, nonadherent cells were washed off from flasks and adherent cells were cultured in Dulbecco’s Modified Eagle Medium (DMEM) with 30% FCS (HyClone defined), penicillin (50 U/ml; GIBCO), and streptomycin (0.05 mg/ml; GIBCO) for 1-3 weeks (medium change every 3 days) to near confluence. After trypsin treatment (Trypsin–EDTA 0.05%, 5 min incubation), cells were removed and re-plated at a density of 50 cells/cm2 in DMEM containing 10% FCS, penicillin (50 U/ml;GIBCO) and streptomycin (0.05 mg/ml; GIBCO) into new flasks and grown to confluence. For all differentiation assays, BMSCs were grown to confluence in 60 mm cell culture dishes. For adipogenic differentiation, BMSCs at confluence were cultured with adipogenic induction medium consisting of DMEM containing 4.5g/L glucose, 10% FCS (HyClone defined), 1 μM dexamethasone, 0.2 mM indomethacin, 0.5 mM 3-Isobutyl-1- methyl-xanthin, 0.01 mg/ml insulin (all reagents from Sigma-Aldrich), penicillin (50 U/ml; GIBCO) and streptomycin (0.05 mg/ml; GIBCO) for 21 days with complete medium exchanged every 3 days. For staining of lipid vacuoles, BMSCs were washed with PBS twice and incubated in freshly filtered Oil Red O solution (Sigma-Aldrich) for 30 minutes. For osteogenic differentiation, BMSCs were cultured in osteogenic induction medium consisting of DMEM supplemented with 10% FCS, 0.1 μM dexamethasone, 0.05 μM L-Ascorbic-acid 2 phosphate, 10 mM glycerol 2-phosphate (all reagents from Sigma-Aldrich), penicillin (50 U/ml; GIBCO) and streptomycin (0.05 mg/ml; GIBCO) for 30 days with a complete medium exchange every 3 days. Calcium deposits were detected with von Kossa staining: cell layers were fixed with 10% formalin (Sigma-Aldrich) for 1 h, incubated with 2% silver nitrate solution (w/v, Sigma-Aldrich) for 10 min in the dark, washed thouroughly with deionized water, and then exposed to bright light for 15 min.

**Analysis of mice.**

To obtain peripheral blood cells and plasma, mice were bled from the retro orbital venous sinus under anaesthesia or by tail bleeding. WBCs, RBCs, and platelets (PLTs) were counted on a scil Vet abc animal blood counter (Viernheim, Germany). Single-cell suspensions from organs were prepared and RBCs were lysed using ammonium chloride. For FACS analysis of BM and spleen MKs, the following antibodies were used: CD3e (145-2C11), CD4 (GK1.5), CD8a (53-6.7), CD19 (MB19-1), Gr-1 (RB6-85C), Ter119 (Ter119), CD45R (RA3-6B2), NK 1.1 (PK136), CD41 (eBioMWReg30), CD61 (209.G3) and CD45.1 (A20) (all eBioscience, San Diego, CA, USA). Staining for mouse stem and progenitor cells was performed using the following antibodies: CD3e (145-2C11), CD4 (GK1.5), CD8a (53-6.7), B220 (RA3-6B2), CD19 (MB19-1), CD11b (M1/70), Gr-1 (RB6-85C), Ter119 (Ter119) and CD127 (A7R34), c-Kit (2B8), Sca-1 (D7), CD34 (RAM34; all eBioscience, San Diego, CA, USA) and CD16/32 (FcgRII/III) (2.4G2; Becton Dickinson, Franklin Lakes, NJ, USA). STAT3-YFP knockin mice were generated by targeting the STAT3 gene by homologous recombination in analogy to the procedure described in Maritano et al. [4]. Homozygous STAT3-YFP knockin mice express a functional fusion protein of the STAT3 α-isoform and yellow fluorescent protein (YFP) characterized earlier [5] under control of the endogenous STAT3 promoter.

References:

1. Irizarry RA, Hobbs B, Collin F et al. Exploration, normalization, and summaries of

high density oligonucleotide array probe level data. Biostatistics (Oxford, England). 2003;4:249-264.

2. Emig D, Salomonis N, Baumbach J et al. AltAnalyze and DomainGraph: analyzing

and visualizing exon expression data. Nucleic acids research. 2010;38:W755-762.

3. Zambon AC, Gaj S, Ho I et al. GO-Elite: a flexible solution for pathway and ontology over-representation. Bioinformatics (Oxford, England). 2012;28:2209- 2210.

4. Maritano D, Sugrue ML, Tininini S et al. The STAT3 isoforms alpha and beta have

unique and specific functions. Nature immunology. 2004;5:401-409.

5. Herrmann A, Sommer U, Pranada AL et al. STAT3 is enriched in nuclear bodies.

Journal of cell science. 2004;117:339-349.

**Supplemental online materials**

Supplemental Figure 1: Tissue expression of G-csf and Angptl4 mRNA transcripts after in vivo LPS stimulation.

(A) G-csf and Angptl4 mRNA expression in the liver, lung and spleen of PBS (white bars) or LPS-treated (grey bars) WT mice. Expression levels are normalized against 18S RNA. Mice were i.p. injected once with 50 μg LPS and analyzed 8 hours later. N.d., not detectable within 35 cycles of amplification. Mean ± SEM of three different experiments each with a total of three PBS- and three LPS- injected mice/group are shown. Statistically significant differences are indicated (*p<0.05, **p<0.01, ***p<0.001).

**Supplemental Figure 2:** Angptl4 is upregulated after Streptococcus pneumoniae infection.

**(A)** Representative FACS profile of Gr-1lowCD11blow/+ and Gr-1highCD11blow/+ cells in the BM of WT mice at 72 h after double PBS or LPS injection (50 μg from 1:1 mixture of E. coli strain K12 and S. minnesota strain R595) or at 48 after Streptococcus pneumoniae (strain D39) or saline injection through the frontolateral skull. **(B)** Blood- and BM-plasma G-CSF and Angptl4 protein levels in saline (white bars) or Streptococcus pneumoniae (grey bars) injected WT mice, treated as in A. Mean ± SEM of two different experiments with a total of five saline and five S.p.–treated mice are shown. Statistically significant differences are indicated (*p<0.05, **p<0.01, ***p<0.001).

**Supplemental Figure 3: Phenotypic and functional and characterization of murine BMSC.**

**(A)** In vitro differentiation of murine BMSCs into adipocytes and osteoblasts. Oil Red O staining of lipid vacuoles was performed 21 days after beginning of stimulation. Von Kossa staining of secreted Ca2+ deposits was done 30 days after initiation of stimulation. One representative out of three independent experiments with different bone marrow donors is shown. **(B)** Immunophenotype of murine BMSCs. Cells were labeled with monoclonal antibodies specific for the molecules indicated (filled histograms, red) and respective isotype controls (filled histograms, yellow); overlays are indicated in orange. One representative analysis out of three independent experiments with different bone marrow donors is shown.

**Supplemental Figure 4:**

**(A) Left:** triglyceride levels in plasma of PBS vs. Angptl4 injected mice. Results of two different experiments with a total of 5 PBS and 5 Angptl4 injected animals are shown. **Right:** BM-plasma Angptl4 protein levels in PBS-treated (white bars) or LPS-treated (grey bars) WT mice. **(B)** Expression of the paired immunoglobulin-like receptor (PIRB) on MK lineage cells. c-kit^+^Sca-1^−^CD150^+^ CD41^+^lin^-^ fraction of the BM. This fraction has previously been shown to contain MK progenitor cells. About 50% of this fraction express the paired immunoglobulin-like receptor (PIRB), which serves as a receptor for Angptl4. **(C)** IL-6 levels in supernatants of cytokine supplemented cultures in in SCF, SCF+TPO, SCF+Angptl4, and SCF+TPO+Angptl4 supplemented cultures at Day 9 after initiating cultures from 1x10^5^ lin- BM cellsin A. Mean ± SEM of three different experiments with a total of 6 SCF, 6 SCF and TPO, 6 SCF and Angptl4, and 6 SCF, Angptl4, and TPO supplemented cultures are shown.

**Supplemental Figure 5: STAT3 and GATA-1 protein expression in megakaryocyte cultures.**

**(A)** Relative expression of stat3 in total cells developed from WT or STAT3-YFP knockin mice in SCF and TPO supplemented cultures at Day 5 of culture. **(B)** Frequency of stat3 expressing CD61+CD41^low/negative^ and CD61+CD41^high^ in SCF, SCF+TPO, SCF+Angptl4, and SCF+TPO+Angptl4 supplemented cultures at Day 5 after initiating cultures from 1x10^5^ lin- BM cells. **(C)** Analysis of STAT3 expression by confocal microscopy at Day 5 in megakaryocytes developed from stat3-YFP knockin mice. Nuclei were stained with DRAQ5. Scale bars represent 10 µm. **(D)** GATA-1 and STAT3 expression in SCF+Angptl4 (upper panel: “immature”) and SCF+Angptl4+TPO (lower panel: “mature”) supplemented cultures at Day 5 after initiating cultures from 1x10^5^ lin- BM cells. Cells were fixed, stained for the respective transcription factors and analysed by confocal microscopy. Nuclei were stained with DRAQ5. Scale bars represent 20 µm.
