# Supplementary material for: Angptl4 is upregulated under inflammatory conditions in the bone marrow of mice, expands myeloid progenitors, and accelerates reconstitution of platelets after myelosuppressive therapy
Source: J Hematol Oncol. 2015 Jun 9;8:64. doi: 10.1186/s13045-015-0152-2 (PMC4460974; doi:10.1186/s13045-015-0152-2)
Supplement: Additional file 2: — Supplementary Figure. [file 13045_2015_152_MOESM2_ESM.pptx]

## Slide 1
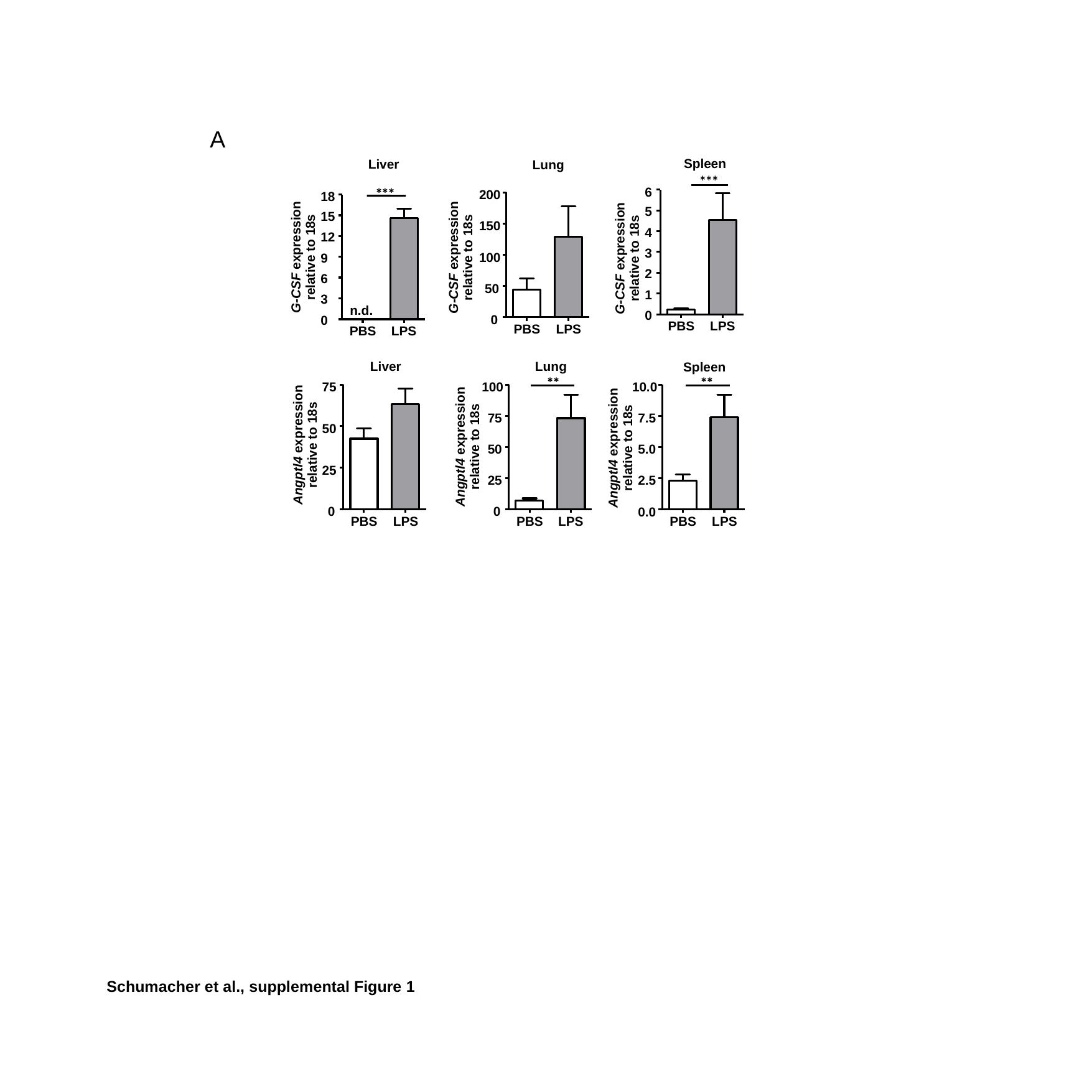

A
Liver
Spleen
Lung
∗∗∗
∗∗∗
6
5
4
3
2
1
0
200
18
15
12
9
6
3
0
n.d.
150
G-CSF expression
relative to 18s
G-CSF expression
relative to 18s
100
G-CSF expression
relative to 18s
50
0
PBS
LPS
PBS
LPS
PBS LPS
Lung
Liver
Spleen
∗∗
∗∗
75
100
10.0
75
7.5
50
Angptl4 expression
relative to 18s
Angptl4 expression
relative to 18s
Angptl4 expression
relative to 18s
50
5.0
25
25
2.5
0
0
0.0
PBS
LPS
PBS LPS
PBS
LPS
Schumacher et al., supplemental Figure 1

## Slide 2
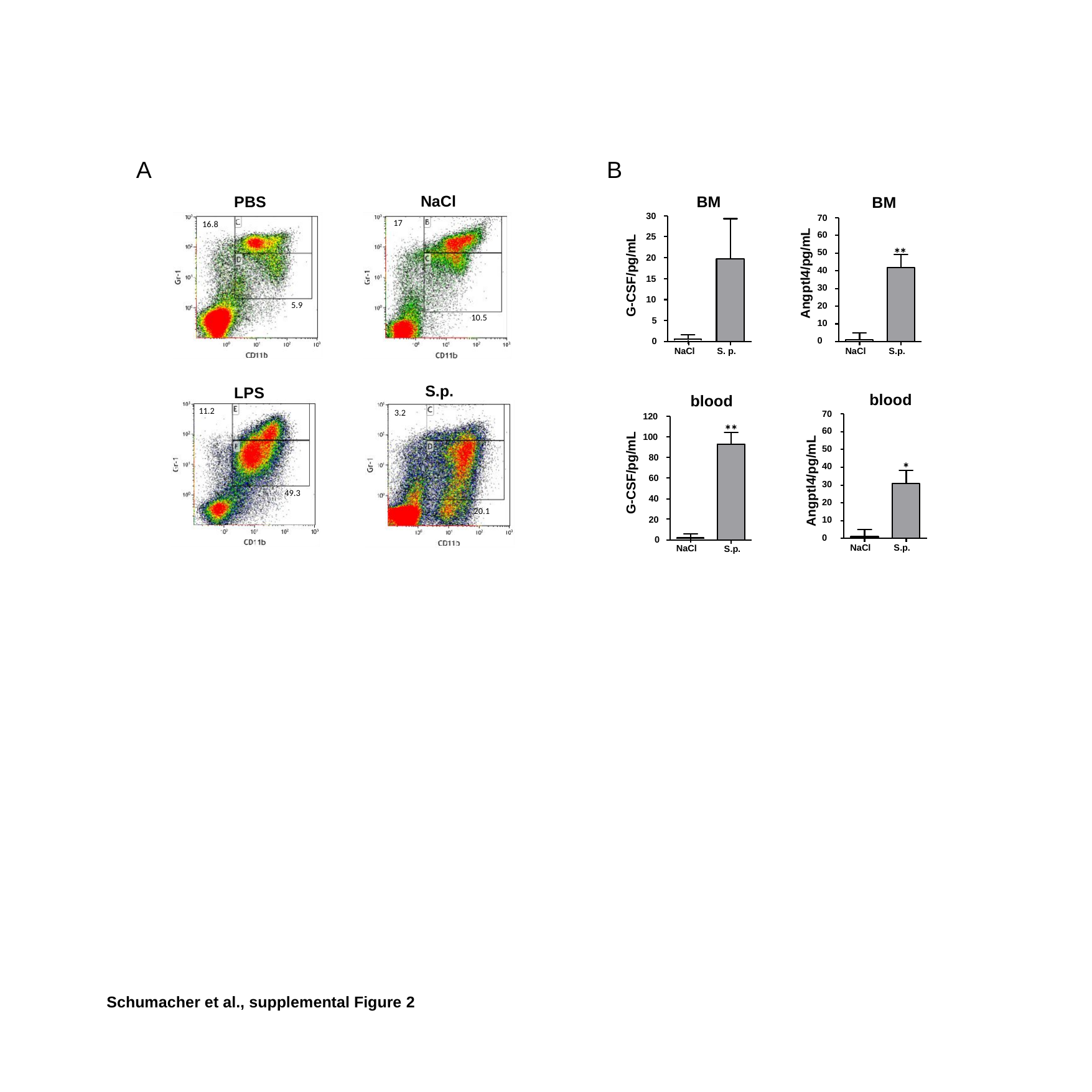

A
B
NaCl
PBS
BM
BM
30
70
60
50
40
30
20
10
0
17
16.8
25
∗∗
20
Angptl4/pg/mL
G-CSF/pg/mL
15
10
5.9
10.5
5
0
NaCl
S. p.
NaCl
S.p.
S.p.
LPS
blood
blood
11.2
3.2
70
60
50
40
30
20
10
0
120
∗∗
100
80
∗
G-CSF/pg/mL
60
Angptl4/pg/mL
49.3
40
20.1
20
0
NaCl S.p.
NaCl
S.p.
Schumacher et al., supplemental Figure 2

## Slide 3
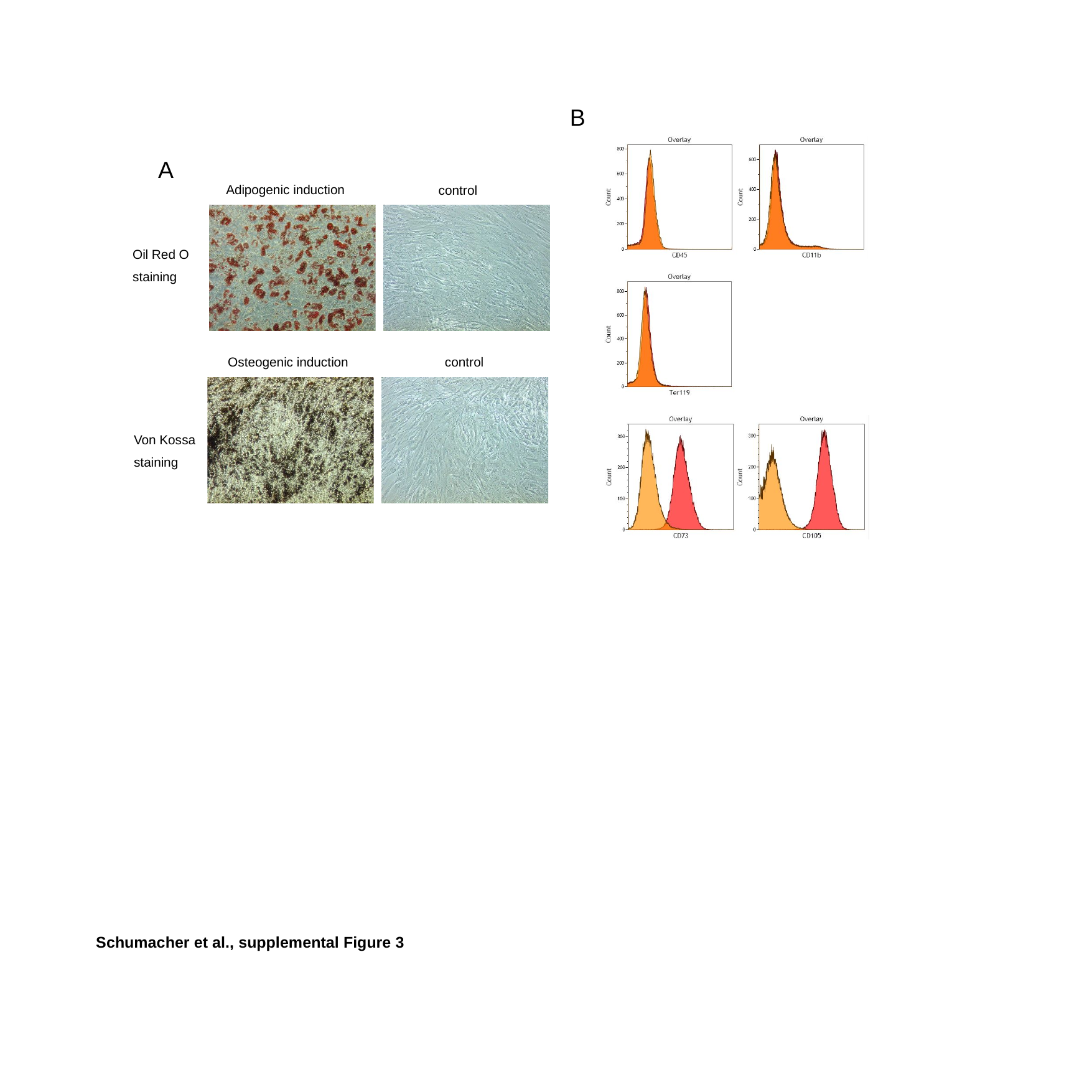

B
A
Adipogenic induction
control
Oil Red O
staining
Osteogenic induction
control
Von Kossa
staining
Schumacher et al., supplemental Figure 3

## Slide 4
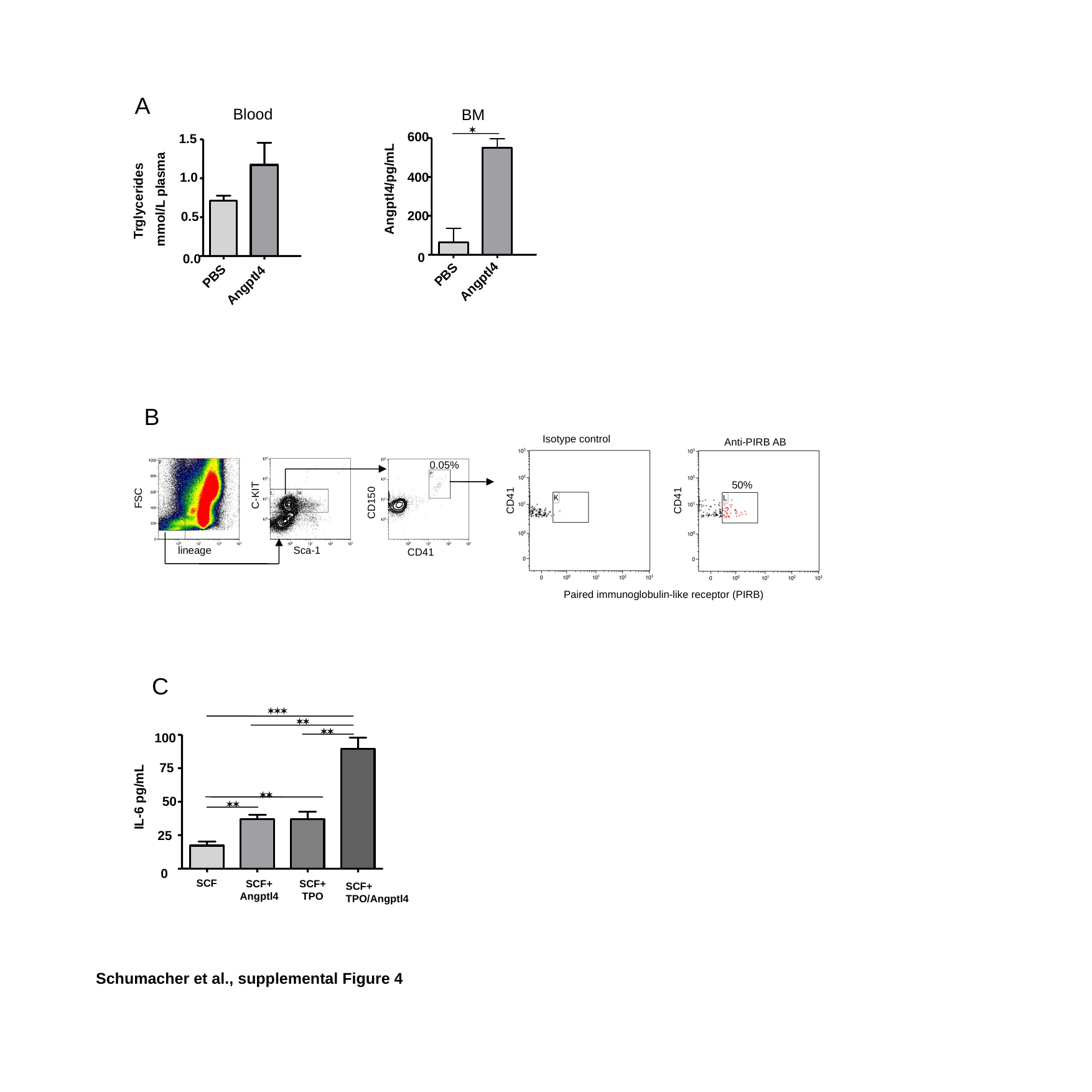

A
Blood
BM

1.5
1.0
Trglycerides
 mmol/L plasma
0.5
0.0
PBS
Angptl4
600
400
Angptl4/pg/mL
200
0
PBS
Angptl4
B
Isotype control
Anti-PIRB AB
0.05%
C-KIT
50%
CD150
CD41
CD41
FSC
Sca-1
lineage
CD41
Paired immunoglobulin-like receptor (PIRB)
C



100
75
IL-6 pg/mL


50
25
0
SCF
SCF+
Angptl4
SCF+
TPO
SCF+
TPO/Angptl4
Schumacher et al., supplemental Figure 4

## Slide 5
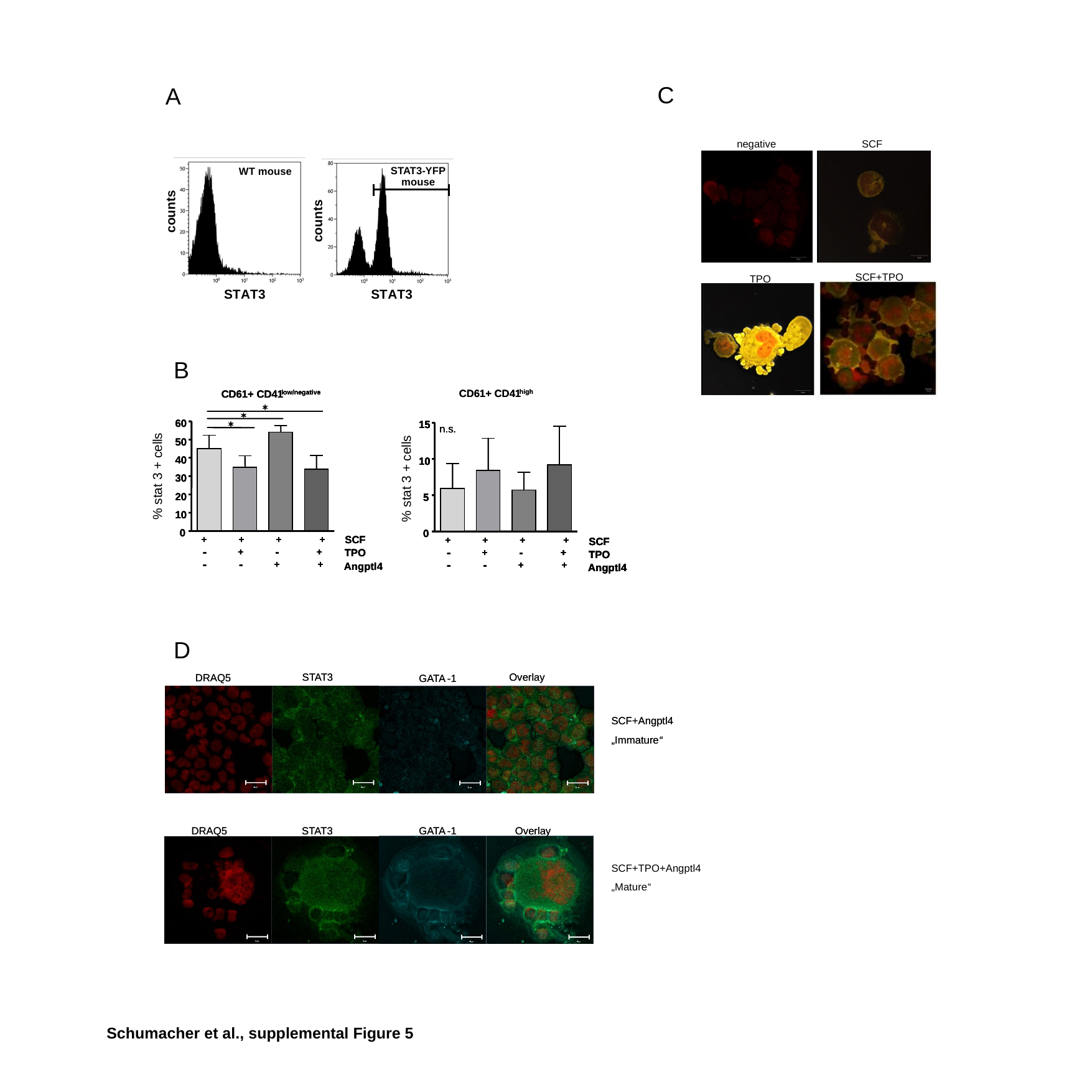

C
A
negative
SCF
STAT3-YFP
mouse
WT mouse
SCF+TPO
counts
counts
SCF+TPO
TPO
STAT3
STAT3
B
CD61+ CD41
CD61+ CD41
high
high
15
10
5
0
+ + + +
SCF
TPO
Angptl4
-
+
-
+
-
-
+ +
n.s
.
15
10
5
0
+ + + +
SCF
TPO
Angptl4
-
+
-
+
-
-
+ +
15
15
n.s
n.s
.
.
10
10
5
5
0
0
+ + + +
+ + + +
SCF
TPO
Angptl4
SCF
SCF
-
-
+
+
-
-
+
+
TPO
TPO
-
-
-
-
+ +
+ +
Angptl4
Angptl4
CD61+ CD41
CD61+ CD41
CD61+ CD41
low/negative
low/negative
low/negative
*
*
*
*
*
*
*
*
*
60
60
60
50
50
50
40
40
40
% stat 3 + cells
30
30
30
20
20
20
10
10
10
0
0
0
+ + + +
+ + + +
+ + + +
SCF
TPO
Angptl4
SCF
SCF
-
-
-
+
+
+
-
-
-
+
+
+
TPO
TPO
-
-
-
-
-
-
+ +
+ +
+ +
Angptl4
Angptl4
% stat 3 + cells
D
Overlay
Overlay
STAT3
STAT3
DRAQ5
DRAQ5
GATA
GATA
-
-
1
1
SCF+Angptl4
SCF+Angptl4
„
„
Immature
Immature
“
“
Overlay
Overlay
DRAQ5
DRAQ5
STAT3
STAT3
GATA
GATA
-
-
1
1
SCF+TPO+Angptl4
„
Mature
“
Schumacher et al., supplemental Figure 5
